# Leukocytoclastic Vasculitis Presenting With Peripheral Neuropathy and Foot Drop in an Adolescent: A Case Report

**DOI:** 10.7759/cureus.114455

**Published:** 2026-08-13

**Authors:** Prasanna Kumar Reddy, Philo Hazeena, Rithvik Ramesh, Leena Dennis Joseph, Lakshmi Narasimhan Ranganathan, Sundar Shanmugam

**Affiliations:** 1 Neurology, Sri Ramachandra Institute of Higher Education and Research, Chennai, IND; 2 Pathology, Sri Ramachandra Institute of Higher Education and Research, Chennai, IND

**Keywords:** cutaneous small-vessel vasculitis, foot drop, leukocytoclastic vasculitis, palpable purpura, sensorimotor neuropathy, vasculitic neuropathy

## Abstract

Leukocytoclastic vasculitis (LCV) is a small-vessel vasculitis that typically presents with palpable purpura on the lower extremities and is often confined to the skin. Peripheral nerve involvement is uncommon. We describe the case of a 17-year-old female who developed progressive burning paresthesias in all four limbs, asymmetric sensory loss, autonomic symptoms, and left foot drop preceding the appearance of palpable purpura and necrotic lesions on both lower limbs. Nerve conduction studies demonstrated mixed demyelinating and axonal motor neuropathy in both lower limbs, more pronounced on the left, with additional motor axonal involvement of the right median nerve. Sympathetic skin responses were absent in all four limbs.

An extensive evaluation for infectious, connective-tissue disease-related, antineutrophil cytoplasmic antibody-associated, and paraproteinemic causes was unrevealing. Skin punch biopsy demonstrated dermal perivascular inflammation, leukocytoclasis, erythrocyte extravasation, and fibrinoid necrosis of small-vessel walls; direct immunofluorescence was positive for complement component 3 (C3), immunoglobulin M (IgM), and IgA. These findings were consistent with LCV. The patient was treated with intravenous methylprednisolone followed by oral corticosteroids and hydroxychloroquine, along with two doses of rituximab. The patient demonstrated marked improvement in left ankle dorsiflexion, with Medical Research Council (MRC) muscle power improving from 2/5 to 4+/5, and was able to walk independently without assistance. No new skin lesions developed. This report highlights that neurologic manifestations may precede the characteristic cutaneous eruption of LCV and that early electrophysiologic assessment and tissue biopsy can facilitate prompt diagnosis and treatment.

## Introduction

Leukocytoclastic vasculitis (LCV), also termed hypersensitivity angiitis, is a cutaneous small-vessel vasculitis affecting dermal capillaries and postcapillary venules [1]. Histopathologic features classically include neutrophilic infiltration of vessel walls, leukocytoclasia, red blood cell extravasation, and fibrinoid necrosis [1]. Cutaneous LCV appears to affect both sexes equally and can occur at any age, although some studies have noted a slight predilection for males and older individuals [2]. In adults, LCV is more commonly associated with an underlying systemic vasculitis, connective tissue disease, or malignancy [2].

The etiology of LCV is estimated to be 45-55% idiopathic, 15-20% infectious, 15-20% related to autoimmune connective tissue diseases or inflammatory disorders, 10-15% drug-induced hypersensitivity reactions, and 5% associated with malignancy or lymphoproliferative disorders [2,3]. The clinical spectrum ranges from skin-limited palpable purpura to systemic disease involving the kidneys, gastrointestinal tract, lungs, joints, or peripheral nervous system [4,5]. Peripheral nerve involvement is uncommon but clinically important [6]. Inflammation of the vasa nervorum may cause ischemic nerve injury, presenting as painful sensory neuropathy, asymmetric sensorimotor neuropathy, mononeuritis multiplex, autonomic dysfunction, or foot drop [6].

While LCV typically presents with palpable purpura as the initial manifestation, neurologic symptoms can rarely precede the characteristic cutaneous eruption, posing a diagnostic challenge and potentially leading to delayed or alternative diagnoses. Diagnosing vasculitic neuropathy before the onset of skin lesions often requires a high index of clinical suspicion and may necessitate biopsy of the skin, even when it appears normal, or of nerve structures to confirm vasa nervorum inflammation [7]. We report an adolescent patient in whom progressive asymmetric neuropathic symptoms and autonomic dysfunction preceded the appearance of the characteristic purpuric eruption.

## Case presentation

A 17-year-old female with no known comorbidities presented with a four-week history of burning pain in both feet accompanied by pins-and-needles sensations. The symptoms initially began on the soles, extended to the dorsum of the feet and ankles, and subsequently involved the dorsal aspects of both hands over a period of four to six days. The symptoms were insidious and progressive and were partially relieved by walking. Approximately 7-10 days after the onset of sensory symptoms, she developed acute-onset purpuric lesions over the dorsum and soles of both feet. The lesions gradually progressed proximally to the knees, with some developing central black discoloration suggestive of evolving necrosis. During the same period, she also noted reduced sweating and hair loss over both lower limbs. Progressive numbness subsequently developed in both lower limbs, more pronounced on the left, extending from the feet to below the knees.

**Figure 1:**
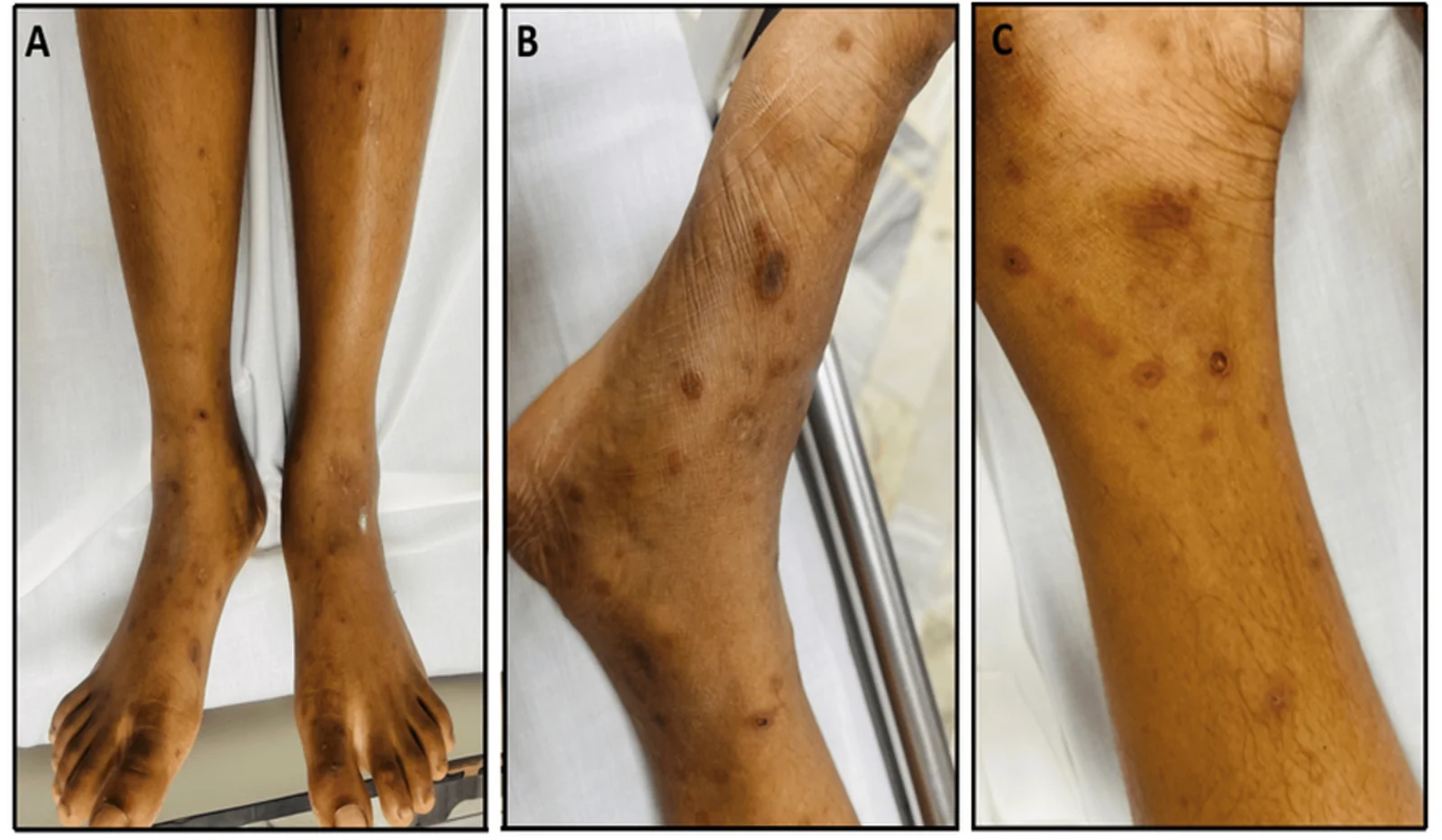
Pretreatment clinical manifestations of leukocytoclastic vasculitis Multiple bilaterally distributed palpable purpuric lesions over the legs, ankles, and dorsum of the feet (A). Several lesions show central dusky discoloration and evolving necrosis, with surrounding post-inflammatory hyperpigmentation (B, C)

One week after the onset of the purpuric skin lesions, she developed progressive difficulty in walking, with impaired left foot clearance, difficulty retaining slippers, and recurrent tripping on both even and uneven surfaces, consistent with an evolving left foot drop. She presented to the Neurology Department two weeks after the onset of motor weakness. There was no history of falls, bowel or bladder dysfunction, recent trauma, preceding infection, toxin exposure, or initiation of any new medication, and no clinical features suggestive of an underlying connective-tissue disorder. Examination showed multiple palpable petechial and purpuric lesions over both legs and feet; several lesions had central necrosis and residual hyperpigmentation (Figures 1A-1C). The skin over the extremities appeared indurated, and the nails were brittle.

Initial sensory examination revealed hyperalgesia to touch and pressure below both knees. On subsequent examination, graded sensory loss was noted below the knees, more pronounced on the left. Motor examination demonstrated left ankle dorsiflexion weakness, graded 2/5 on the Medical Research Council (MRC) scale, with a corresponding left-sided high-stepping gait. Deep tendon reflexes in the lower limbs were reduced, with knee jerks graded as 1+ bilaterally and ankle jerks absent bilaterally. Plantar responses were down-going on both sides. Loss of hair and abnormal sweating over both lower limbs suggested associated autonomic involvement. Romberg testing was positive, and tandem gait was impaired.

Investigations

The evaluation was directed toward a distal-predominant, asymmetric sensorimotor neuropathy in association with purpuric skin lesions. Routine hematologic and biochemical investigations, including complete blood count, renal and liver function tests, thyroid function tests, and glycemic profile, were within reference limits. However, the erythrocyte sedimentation rate and C-reactive protein levels were elevated, indicating an inflammatory response. Serum immunoglobulin E (IgE) was also increased. The absolute eosinophil count, serum IgA, creatine phosphokinase, lactate dehydrogenase levels, and vitamin B12, complement component 3 (C3), and C4 levels remained within their respective reference ranges. The relevant laboratory findings are summarized in Table 1, while Table 2 summarizes the investigations performed to identify secondary causes.

**Table 1:** Summary of principal laboratory findings obtained during the diagnostic workup CRP: C-reactive protein; CPK: creatine phosphokinase; ESR: erythrocyte sedimentation rate; IgA: immunoglobulin A; IgE: immunoglobulin E; LDH: lactate dehydrogenase

Laboratory parameter	Patient result	Reference range	Interpretation
ESR	28 mm/hr	< 20 mm/hr	Increased
CRP	21 mg/L	< 5 mg/L	Increased
Absolute eosinophil count	130 cells/mm³	40-500 cells/mm³	Within reference range
Serum IgE	> 2,500 IU/mL	< 100 IU/mL	Increased
Serum IgA	216 mg/dL	60-310 mg/dL	Within reference range
CPK	93 U/L	20-180 U/L	Within reference range
LDH	173 U/L	135-214 U/L	Within reference range

**Table 2:** Summary of investigations for secondary causes ANA: antinuclear antibody; anti-CCP: anti-cyclic citrullinated peptide; ANCA: antineutrophil cytoplasmic antibody; ASO: antistreptolysin O; HRCT: high-resolution computed tomography

Domain	Test(s)	Result
Autoimmune/vasculitis	ANA by immunofluorescence, rheumatoid factor, anti-CCP antibody, line immunoassay, c-ANCA, p-ANCA, and ASO titer	Negative
Infectious	Viral serology and fever panel, including Leptospira and scrub typhus	Negative
Paraproteinemia	Serum protein electrophoresis	No paraprotein band
Cardiopulmonary/abdominal imaging	Two-dimensional echocardiography, abdominal ultrasonography, and HRCT chest	No significant abnormality; no chest mass, consolidation, interstitial lung disease, or mediastinal lymphadenopathy

Nerve conduction studies demonstrated a mixed demyelinating and axonal motor neuropathy involving both lower limbs, with greater involvement on the left, along with motor axonal neuropathy of the right median nerve. Autonomic function testing showed absent sympathetic skin responses in both the upper and lower limbs, whereas R-R interval testing demonstrated preserved parasympathetic function. The lysosomal group-1 enzyme assay was negative.

**Figure 2:**
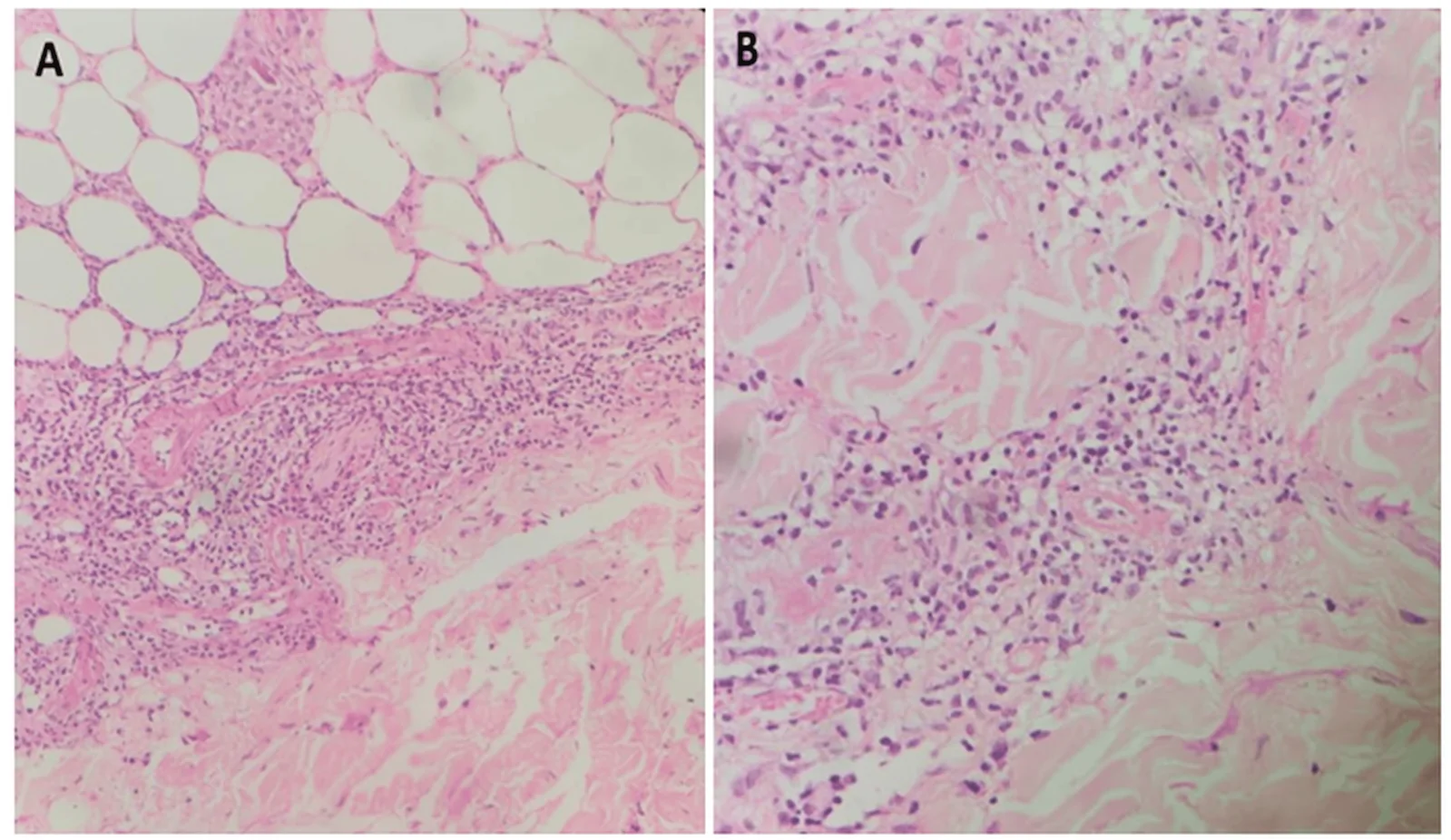
Histopathologic findings from the left-leg skin punch biopsy Hematoxylin and eosin-stained sections demonstrate (A) dense perivascular inflammatory infiltration involving small dermal vessels, with extravasated red blood cells and focal fibrinoid necrosis of the vessel wall. (B) A higher-magnification view of the involved dermal vessels and surrounding inflammatory cells. These findings support the diagnosis of cutaneous small-vessel vasculitis

Given the combination of painful asymmetric neuropathy, autonomic dysfunction, left foot drop, and purpuric skin lesions, vasculitic neuropathy was clinically suspected. A skin punch biopsy was obtained from a fresh, active purpuric lesion on the left leg. Histopathological examination showed an unremarkable epidermis. The upper and deep dermis demonstrated a perivascular lymphocytic inflammatory infiltrate admixed with occasional plasma cells and extravasated erythrocytes. A few small dermal vessels showed fibrinoid necrosis of the vessel walls, leukocytoclasia, interstitial inflammatory infiltrates, and red blood cell extravasation. The inflammatory infiltrate extended through the deep dermis into the subcutaneous tissue (Figures 2A-2B). Direct immunofluorescence demonstrated deposition of C3, IgM, IgA, and fibrinogen. The combined histopathological and immunofluorescence findings were consistent with LCV.

Treatment and outcome

**Figure 3:**
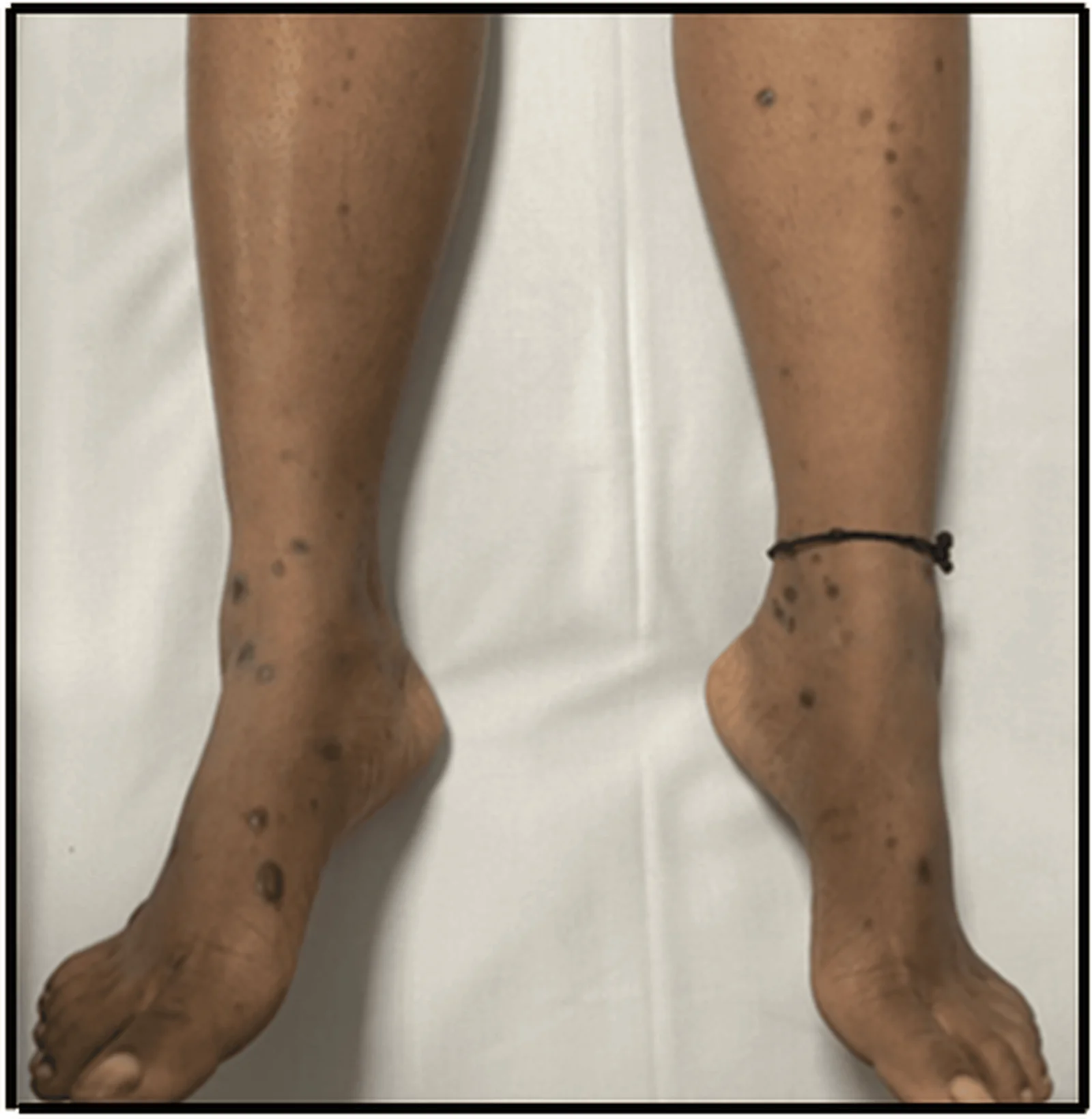
Post-treatment clinical photograph showing resolution of the active purpuric lesions, with residual hyperpigmentation and no new lesions

The patient received intravenous methylprednisolone 1 g once daily for five days, followed by oral corticosteroids and hydroxychloroquine. She also received rituximab 1 g intravenously in two doses administered 15 days apart. Following treatment, the paresthesias and left ankle dorsiflexion weakness improved substantially, with muscle strength increasing from MRC grade 2/5 at presentation to 4+/5 after treatment. She was able to ambulate independently without support, and no new skin lesions developed (Figure 3).

## Discussion

LCV results from inflammation of small vessels, most often the postcapillary venules of the skin [1]. Immune-complex deposition may activate complement and recruit inflammatory cells, leading to endothelial injury, vessel-wall necrosis, red blood cell extravasation, and fragmentation of inflammatory-cell nuclei [1]. Clinically, this process most commonly produces palpable purpura in dependent areas such as the legs and feet [1]. LCV may be idiopathic or secondary to infection, medications, malignancy, connective tissue disease, immune-complex disorders, or systemic vasculitis [2,3]. Distinguishing skin-limited disease from systemic involvement is important because renal, gastrointestinal, pulmonary, articular, and neurologic manifestations may require broader evaluation and more intensive treatment [4].

In our case, autoimmune and systemic vasculitic causes were evaluated with antinuclear antibody (ANA), rheumatoid factor, anti-cyclic citrullinated peptide (anti-CCP) antibodies, line immunoassay, cytoplasmic antineutrophil cytoplasmic antibody (c-ANCA), and perinuclear-ANCA (p-ANCA). These investigations were unrevealing, thereby reducing the likelihood of connective-tissue disease-associated vasculitis and ANCA-associated vasculitides, including granulomatosis with polyangiitis and microscopic polyangiitis. However, we recognize that negative ANCA testing does not by itself completely exclude ANCA-associated vasculitis, particularly in limited disease. Complement C3 and C4 levels were also assessed to look for evidence of complement-consuming immune-complex disease.

The infectious evaluation included viral serology and a fever panel, including testing for Leptospira and scrub typhus, together with an antistreptolysin O (ASO) titre to assess for a possible antecedent streptococcal trigger. No supporting evidence of an infectious precipitant was identified. In addition, the patient had no history of a recent infection, toxin exposure, or introduction of a new medication, making an obvious infection- or drug-triggered hypersensitivity vasculitis less likely. Paraproteinemic and hematologic causes were considered because monoclonal gammopathies and lymphoproliferative disorders may be associated with both cutaneous vasculitis and peripheral neuropathy. Serum protein electrophoresis demonstrated no paraprotein band, making a monoclonal gammopathy-related process less likely.

Routine hematological parameters were also within reference limits. Renal and hepatic function were normal, and cardiopulmonary and abdominal evaluation, including two-dimensional echocardiography, abdominal ultrasonography, and high-resolution CT (HRCT) of the chest, showed no significant abnormality. In particular, HRCT demonstrated no pulmonary mass, consolidation, interstitial lung disease, or mediastinal lymphadenopathy. These findings provided no evidence of clinically apparent pulmonary or major systemic organ involvement. Thyroid and glycemic profiles were also normal, helping to exclude common metabolic causes of peripheral neuropathy, while normal creatine phosphokinase made a primary myopathic process less likely.

Taken together, the clinical history, laboratory investigations, and imaging did not identify a convincing secondary systemic cause for the vasculitis. Importantly, the diagnosis itself was supported by tissue examination: skin punch biopsy demonstrated small-vessel wall injury with fibrinoid necrosis, leukocytoclasia, and erythrocyte extravasation, while direct immunofluorescence demonstrated C3, IgM, IgA, and fibrinogen deposition. These findings were consistent with leukocytoclastic small-vessel vasculitis. Continued clinical follow-up remains important, as an underlying systemic disorder may occasionally become apparent later in the disease course.

Neuropathy associated with vasculitis is generally caused by inflammatory injury to the vasa nervorum and is typically painful, asymmetric, and axonal, although mixed electrophysiologic patterns may occur [6,7]. The combination of burning pain, asymmetric sensory loss, foot drop, sluggish reflexes, and absent sympathetic skin responses strongly suggested peripheral nerve and autonomic involvement in this case [6]. The additional right median motor axonal neuropathy indicated a multifocal process rather than an isolated common peroneal neuropathy.

The temporal sequence is the principal learning point. Neuropathic symptoms began approximately one week before the purpuric eruption, potentially delaying recognition of an underlying vasculitic process [7]. When painful, asymmetric neuropathy or acute foot drop is accompanied by evolving purpura, necrosis, or autonomic skin changes, early dermatologic examination, electrophysiologic testing, and biopsy of a fresh lesion should be considered [7,8,9]. A nerve or combined nerve-muscle biopsy may provide more direct evidence of vasculitic neuropathy when the diagnosis remains uncertain or when treatment decisions depend on histologic confirmation [8,9].

The patient was initially treated with intravenous methylprednisolone 1 g once daily for five days, followed by oral corticosteroids. Hydroxychloroquine was subsequently added as an adjunctive immunomodulatory agent in view of the suspected immune-mediated nature of the vasculitis and the inadequate initial response to pulse corticosteroid therapy [1,8,9]. Despite this regimen, new cutaneous lesions continued to appear, and motor weakness showed no substantial improvement, although the paresthesias decreased [8,9]. Given the persistent disease activity and inadequate clinical response, rituximab therapy was initiated [8]. She received two intravenous doses of rituximab, 1 g each, administered 15 days apart [8,9]. Following rituximab therapy, there was a marked improvement in left ankle dorsiflexion weakness, from MRC grade 2/5 at presentation to 4+/5 following treatment, with independent ambulation without assistance or a foot-drop splint and no further development of new skin lesions [1,8,9].

## Conclusions

In rare cases, LCV can present with neurological manifestations before the appearance of characteristic palpable purpura. In young patients with painful asymmetric sensorimotor deficits, autonomic dysfunction, or foot drop, careful serial skin examination is therefore important, even when the initial vasculitis workup is unrevealing. Early electrophysiological evaluation and timely biopsy of a fresh, active skin lesion may facilitate early recognition of an underlying small-vessel vasculitic process and enable treatment before irreversible axonal injury develops. Continued long-term clinical follow-up is also essential, as an underlying systemic or autoimmune disorder may become apparent later in the disease course.

## References

[1] Fraticelli P, Benfaremo D, Gabrielli A (2021). Diagnosis and management of leukocytoclastic vasculitis. Intern Emerg Med.

[2] Bouiller K, Audia S, Devilliers H (2016). Etiologies and prognostic factors of leukocytoclastic vasculitis with skin involvement: a retrospective study in 112 patients. Medicine (Baltimore).

[3] Casale JJ, Muse ME, Snow TJ, Gould KP, Depcik-Smith ND (2022). Leukocytoclastic vasculitis following the first dose of the elasomeran COVID-19 vaccination. Case Rep Dermatol Med.

[4] Pantic I, Jevtic D, Nordstrom CW, Madrid C, Milovanovic T, Dumic I (2022). Clinical manifestations of leukocytoclastic vasculitis, treatment, and outcome in patients with ulcerative colitis: a systematic review of the literature. J Clin Med.

[5] Malik MJ, Pasha MN, Salib V (2023). Leukocytoclastic vasculitis: a case report. Cureus.

[6] Ferranti M, Cama E, Cacciavillani M, Schiavon F, Felicetti M, Briani C, Alaibac M (2021). Leukocytoclastic vasculitis associated with multifocal sensory neuropathy responsive to intravenous immunoglobulins: a case report. Sarcoidosis Vasc Diffuse Lung Dis.

[7] Yamanaka Y, Hiraga A, Arai K (2006). Leucocytoclastic vasculitic neuropathy diagnosed by biopsy of normal appearing skin. J Neurol Neurosurg Psychiatry.

[8] Sunderkötter C, Bonsmann G, Sindrilaru A, Luger T (2005). Management of leukocytoclastic vasculitis. J Dermatolog Treat.

[9] Alpsoy E (2022). Cutaneous vasculitis; an algorithmic approach to diagnosis. Front Med (Lausanne).

